# Enhanced Antibacterial Activity of *Echinacea angustifolia* Extract against Multidrug-Resistant *Klebsiella pneumoniae* through Niosome Encapsulation

**DOI:** 10.3390/nano11061573

**Published:** 2021-06-15

**Authors:** Maryam Moghtaderi, Amir Mirzaie, Negar Zabet, Ali Moammeri, Amirreza Mansoori-Kermani, Iman Akbarzadeh, Faten Eshrati Yeganeh, Arman Chitgarzadeh, Aliasghar Bagheri Kashtali, Qun Ren

**Affiliations:** 1 School of Chemical Engineering, College of Engineering, University of Tehran, Tehran 1417935840, Iran; mmoghtaderi90@gmail.com (M.M.); moameriali21@gmail.com (A.M.); 2 Department of Biology, Parand Branch, Islamic Azad University, Parand 3761396361, Iran; 3 Department of Medicinal Chemistry, School of Pharmacy, Shahid Beheshti University of Medical Sciences, Tehran 1996835113, Iran; zabet.n.orchid@gmail.com; 4 Department of Chemical and Petrochemical Engineering, Sharif University of Technology, Tehran 1458889694, Iran; amirrezamansory@gmail.com; 5 Department of Chemistry, Science and Research Branch, Islamic Azad University, Tehran 1477893855, Iran; ffyeganeh@gmail.com; 6 Department of Biology, Roudehen Branch, Islamic Azad University, Roudehen 3973188981, Iran; armanchitgar@gmail.com (A.C.); alibio81@yahoo.com (A.B.K.); 7 Laboratory for Biointerfaces, Empa, Swiss Federal Laboratories for Materials Science and Technology, 9014 St. Gallen, Switzerland

**Keywords:** *Echinacea angustifolia*, niosome, encapsulation, antibacterial activity, stability, drug delivery

## Abstract

With the increased occurrence of antibiotic-resistant bacteria, alternatives to classical antibiotics are urgently needed for treatment of various infectious diseases. Medicinal plant extracts are among the promising candidates due to their bioactive components. The aim of this study was to prepare niosome-encapsulated *Echinacea angustifolia* extract and study its efficacy against multidrug-resistant *Klebsiella pneumoniae* strains. Encapsulation was first optimized by Design of Experiments, followed by the empirical study. The obtained niosomes were further characterized for the size and morphology using dynamic light scattering (DLS), transmission electron microscopy (TEM), and scanning electron microscopy (SEM). Spherical niosomes had a diameter of 142.3 ± 5.1 nm, as measured by DLS. The entrapment efficiency (EE%) of *E. angustifolia* extract reached up to 77.1% ± 0.3%. The prepared niosomes showed a controlled drug release within the tested 72 h and a storage stability of at least 2 months at both 4 and 25 °C. The encapsulated *E. angustifolia* displayed up to 16-fold higher antibacterial activity against multidrug-resistant *K.* *pneumoniae* strains, compared to the free extract. Additionally, the niosome exhibited negligible cytotoxicity against human foreskin fibroblasts. We anticipate that the results presented herein could contribute to the preparation of other plant extracts with improved stability and antibacterial activity, and will help reduce the overuse of antibiotics by controlled release of natural-derived drugs.

## 1. Introduction

Non-ionic surfactants and cholesterol (lipid) could arrange a biologically acceptable structure called niosome [1,2,3], which were first applied in L’Oréal’s cosmetic commodities assignable to their weak excitability potential [4]. Many researchers have explored these colloidal emulsions in novel drug delivery systems for the therapeutic applications due to their excellent specification, such as insolubility, biocompatibility, and capability to carry both hydrophilic and hydrophobic drugs [5,6]. The content of surfactants and lipids can be altered to optimize the formulation size and the drug encapsulation efficiency to achieve high desired activity for each drug [7,8]. Various drugs have been formulated in the form of niosomes. Many of them focus on the encapsulation of conventional antibiotics or metal-based agents, however, these drugs can have toxic side effects, and may even promote the development of antibiotic-resistant bacteria strains due to the sub-inhibitory dose delivery [9].

Due to the increased occurrence of antibiotic-resistant bacteria, which poses a huge risk to humankind and a burden to the society, alternatives to classical antibiotics are imperative. Herbal medicine can be such candidates, which manifest additional advantages, including their light side effects and antimicrobial activity. *Echinacea angustifolia* is a natural wizard that has traditionally been applied to treat many diseases, such as simple cold and cough, toothaches, bowel pain, and wound infections. It has been reported that *E. angustifolia* contains various bioactive compounds, including alkyl amides, polyphenols, caffeic acid prototype, low molecular weight polysaccharides, alkaloids, and lipophilic elements [10]. Previous studies have demonstrated that encapsulation of *E. angustifolia* extract in nanoparticles enhanced its antimicrobial activity. *E*. *angustifolia*-loaded phytosomal and liposomal structures also resulted in intensified antibacterial and antioxidant activity [11,12]. Niosome-based delivery of *E. angustifolia* extracts has not been reported so far, even though utilization of niosomes to deliver plant essential oils have been attempted. It was found that niosome encapsulation of myrtle essential oil increased antimicrobial activity against *Staphylococcus aureus, S. epidermidis*, *Serratia marcescens*, and *Bacillus subtilis,* compared to free myrtle essential oil [13]. *Satureja Montana* essential oil-loaded niosome was reported to possess enhanced activity against *Aspergillus flavus*, relative to the non-encapsulated form [14].

The goal of this work is thus to develop a stable niosome system for encapsulation of *E. angustifolia* extracts to achieve controlled drug release and high antimicrobial activity. *Klebsiella pneumoniae*, one of the multidrug-resistant opportunistic bacteria known as a leading cause of nosocomial infections, was used as the model pathogenic bacteria. Formulations of the encapsulated *E. angustifolia* extract were optimized and studied in terms of encapsulation efficiency, release profile, particle size, and stability features. Moreover, its antibacterial activity and cytotoxicity were evaluated and compared with free *E. angustifolia* extract.

## 2. Materials and Methods

### 2.1. Materials

All chemicals and reagents were purchased with analytical purity from Merck (Darmstadt, Germany) and applied as received unless otherwise noted. Dialysis membrane (MWCO 12,000 Da) was purchased from Merck (Darmstadt, Germany). RNA extraction (Cat No. ER101-01) and cDNA synthesis kits (AE301-02) were purchased from TransGene Biotech Co., Ltd. (Beijing, China). The cell culture-related materials including RPMI-1640, 3-[4,5-Dimethyl-2-thiazolyl]-2,5-diphenyl tetrazolium bromide (MTT), and PBS (phosphate-buffered saline) were attained from Gibco (Grand Island, NY, USA). The human foreskin fibroblast (HFF) cells were provided by the Pasteur Institute of Iran (Tehran, Iran).

### 2.2. Preparation of E. angustifolia Extract

*E. angustifolia* was obtained from the plant bank of the Center for Biological Resources of Iran and approved for research usage by the botanical department, with the number of each barium of 1659. To prepare the extract, the aerial parts of *E. angustifolia* were first placed in the air and then dried completely in the shade. The aerial parts were completely pulverized by a grinder and kept in glass containers. The prepared powder was used for extraction by the maceration method. Briefly, 20 g of powder was added to 200 mL of absolute ethanol solvent and the extraction was performed for 12 h at room temperature. Finally, the solvent was removed by a rotary evaporator (Rv10 digital, Fisher Scientific, IKA, Germany). The obtained solid powder was washed twice with distilled water and was kept at 4 °C until being used for the synthesis of niosomes.

### 2.3. Optimization of Niosomes-Encapsulated E. angustifolia by Design of Experiments

Design of Experiments 10.0.3 software (Stat-Ease Inc., Minneapolis, MN, USA) applying the Box-Behnken methodology was used to investigate the effect of self-governing variables (hydration time, hydration volume, and the cholesterol content; Table 1) on physicochemical features of *E. angustifolia*-loaded niosomes. Table 2 shows these factors, their degree, as well as their impacts on the nanoparticle size and entrapment efficiency (EE). The minimal size of the niosomes and the maximal entrapment efficiency were taken as the optimization criteria based on the multi-criteria optimization used [15]. The data optimization of the D-optimal study was performed based on the desirability index [16]. Furthermore, the discrepancies of the anticipated and the perceived results were computed, and the optimized formation was then discerned for more far-away studies [17,18,19].

The thin-layer hydration method described in our prior research with slight changes was used to prepare the *E. angustifolia*-loaded niosomes [20]. Briefly, Span 60, Tween 60, and cholesterol were suspended in an organic solvent (2:1 of chloroform:methanol (*v*/*v*), 10 mL), accompanied by evaporation of solvents using a rotary evaporator (150 rpm, 60 °C, 30 min). Subsequently, thin layers were hydrated, and 15 mg of the drug (concentration of 1.5 mg mL^−1^) with different hydration volumes and time (Table 1 and Table 2) was added at 60 °C and 120 rpm. Lastly, 7 min of sonication was performed to obtain the uniform size distribution of *E. angustifolia*-loaded niosomes. The specimens were refrigerated (4 °C) for further experimental research. The compositions of niosomal formation are listed in Table 2.

### 2.4. Characterization of the Synthesized Niosomes

#### 2.4.1. Size, Morphology, and Polydispersity Index (PDI)

The mean size and distribution of niosomes were investigated using the dynamic light scattering (DLS) method (Malvern Zetasizer, Malvern Instrument, London, UK). Briefly, the niosomes suspension was diluted (1:100) with deionized water to prevent additional scattering caused by antimatter cooperation. The size and morphology of the synthesized niosomes were also examined by scanning electron microscopy (SEM, NOVA NANOSEM 450 FEI model, Lincoln, CA, USA) and transmission electron microscopy (TEM, Zeiss EM900, Los Altos, CA, USA). The niosomes suspension was added (1:100) to deionized water for SEM imaging. A dash of the specimen was extended on a conveyor film (aluminum, at room temperature to evaporate the water) [21]. For TEM, niosome-encapsulated *E. angustifolia* extract was placed on the carbon film to prepare the sample for imaging [20].

#### 2.4.2. Fourier-Transform Infrared Spectroscopy (FTIR)

The molecular interaction of *E. angustifolia* extract and niosomes was explored by FTIR spectroscopy (Spectrum Two, Perkinelmer, Waltham, MA, USA). To this end, lyophilized examples were individually processed in KBr, and afterward, the pellets were created by putting the specimen in a hydraulic rush. Room temperature FTIR assessment was carried out within the range of 4000 to 400 cm^−1^ and at a resolution of 4 cm^−1^ [20].

#### 2.4.3. Entrapment Efficiency

The non-encapsulated drug departed from drug-loaded niosomes was assessed to determine EE. In a typical procedure, 1 mL of *E. angustifolia*-loaded niosomes was centrifuged (1 h, 14,000× *g*, 4 °C). The amount of *E. angustifolia* in the supernatant was measured by UV-vis spectroscopy (JASCO, V-530, Tokyo, Japan) at a maximum-wavelength absorbance peak of the drug molecule (365 nm) [22]. The EE can be determined by:

$$\mathrm{Entrapment}\;\mathrm{Efficiency}\;(\%)=\left[\left(A-B\right)/A\right]*100$$

where, A is the quantity of primary drug entrapped into the niosomal structures, and B is the non-entrapped drug mass crossing the membrane.

#### 2.4.4. Study of Drug Release

A dialysis bag (MWCO = 12 kDa) was used for studying in vitro drug release. The dialysis bag was placed in the PBS solution (50 mL, 1X, pH = 3, 5, 7.4) under gradual stirring (50 rpm) at 37 °C. Sampling was performed at different time intervals with replacing by fresh PBS. Different patterns of release kinetics (zero-order, first-order, Higuchi, and Korsmeyer–Peppas model) were performed to analyze their release profiles. Likewise, the analysis was performed for drug solutions with similar initial drug concentrations in the same dialysis bag.

#### 2.4.5. Stability Studies

To assess the stability, the optimized niosomes were stored in several parts at 25 ± 1 °C (room temperature) and 4 ± 1 °C (refrigeration temperature) for 2 months. The physical features such as average nanoparticle size (nm) and entrapment efficiency were determined at a specified interval (0, 14, 30, and 60 days).

### 2.5. Bioactivity of the Synthesized Niosomes

#### 2.5.1. Isolation of *K. pneumoniae* Strains and Their Antibiotic Susceptibility

In this experimental study, 100 clinical samples, including blood, wound, pus, sputum, urine, and cerebrospinal fluid (CSF), were collected from different hospitals in Iran, including Pars, Firoozgar, and Gandi, from September 2019 to August 2020. *K. pneumoniae* strains were isolated and identified using microbiological and biochemical methods [23]. The antibiotic susceptibility was also studied using the Kirby–Bauer disk diffusion method based on the CLSI (Clinical and Laboratory Standards Institute, 2019) procedure toward 10 antibiotics, including Ceftazidime (CAZ), Chloramphenicol (C), Gentamicin (GN), Tobramycin (TOB), Impinem (IMP), Amikacin (AK), Tetracycline (TE), Ampicillin (AMP), Ciprofloxacin (CIP), Amoxicillin/clavulanic acid (AMC), Nalidixic acid (NA), and Colistin (CT). Multidrug-resistant (MDR) isolates were selected based on their resistance to at least one antibiotic.

#### 2.5.2. Antibacterial Efficacy

##### Broth Microdilution Assay

Minimum inhibitory concentration (MIC) and minimum bactericidal concentration (MBC) of *E. angustifolia* extract (free, and niosome-encapsulated) were examined using the micro-dilution method based on the CLSI guidelines. Different concentrations of free and encapsulated extract ranging from 62.5 to 4000 μg mL^−1^ were obtained by making dilutions with Müller–Hinton Broth (MHB). MHB was prepared according the manufacturer’s instructions, namely 21 g of medium powder was dissolved in one liter of distilled water and autoclaved for 15 min at 121 °C. First, 200 μL of each concentration was added to a 96-well plate, then 80 μL of MHB and 20 μL of microbial suspension were added at the concentration of 5 × 10^5^ colony forming units (CFU) mL^−1^, followed by 24 h of incubation at 37 °C to determine the MIC and MBC values. MIC is the lowest concentration of the drug capable of inhibiting bacterial growth. To determine the MBC value, 10 μL of suspension in the MIC well was removed and cultured on Müller Hinton agar medium (MHA) for 24 h at 37 °C. MBC refers to the lowest concentration of drug capable of reducing the bacterial population by more than 99.9% compared to drug-free samples. In this test, negative and positive controls were drug-free wells with and without bacteria, respectively.

#### Time Kill Assay

The *K. pneumoniae* strains (10^6^ CFU mL^−1^) were cultured on Müller–Hinton Broth culture medium containing ½ MIC of niosome-encapsulated *E. angustifolia* extract and free *E. angustifolia* for 10 h at 37 °C. At intervals of 0, 2, 4, 8, and 10 h, the growth rate was monitored by reading the absorption at 600 nm and compared with the control sample without drug. Additionally, at the mentioned intervals, 200 μL was sampled, serial dilution was prepared and plated on Tryptic Soy Broth (TSB, Merck, Darmstadt, Germany) agar medium, and the CFU number was enumerated [24]. The results were expressed as log10 CFU mL^−1^ ± SD. All tests were performed in triplicate, and the average and standard deviation were calculated.

#### 2.5.3. Cytotoxicity Study

The cytotoxicity of niosome, free, and encapsulated *E. angustifolia* extracts was evaluated against the HFF normal cell line using MTT assay. The viable HFF cells subjected to distilled water were used as control and set as 100%. The cytotoxic cut-off was set to 70% viable cells, i.e., a lethal dose of 30%. First, 10^5^ cells were seeded in a 96-well plate and treated with different concentrations of extract (250–4000 µg mL^−1^) for 24 h at 37 °C, with 5% CO_2_. Subsequently, 100 μL of MTT dye (0.5 mg mL^−1^) was poured into the wells, and incubation was continued at 37 ° C for 4 h. The supernatant was then removed followed by adding 100 μL of DMSO and shaking for 5 min. Finally, the optical density of the wells was read by an ELISA plate reader at 570 nm, and the cell survival rate was calculated by the formula:

Cell viability (%) = Optical density of sample/Optical density of control × 100

### 2.6. Statistical Analysis

All experiments were performed twice, in triplicate for each test. The results were expressed as average with standard deviation (SD). The statistical analysis was performed by *t*-test and one-way and two-way analysis of variance (ANOVA) using GraphPad Prism software (version 8). *p* < 0.05 was considered as significant.

## 3. Results and Discussion

### 3.1. Fabrication and Optimization of E. angustifolia-Loaded Niosomes

Niosomes with the long alkyl chain (C18) surfactants, such as Span 60 and Tween 60, yield higher entrapment efficiency and are more stable than those with shorter-length surfactants [24]. Cholesterol is one of the compounds often used to make nanocarriers’ membranes [25]. In niosomes, the interaction between cholesterol and surfactant is through the formation of hydrogen bonds between hydroxyl groups and the alkyl chain of surfactant molecules, which can change the fluidity of the chains in two layers, by increasing the transfer temperature of the vesicles and improving the stability [20,25]. It is noteworthy that the surfactants used in this study were generally regarded as safe (GRAS) [26]. Thus, in this work, we applied these known substances to optimize niosome fabrication to achieve good stability and high bioactivity.

All the niosome formations were prepared based on the Design of Experiments described in the Section 2. The effects of several variables including hydration time (A), hydration volume (B), and cholesterol proportion (C) on the entrapment efficiency and the size of niosomes were analyzed and are shown in Table 2. The niosome size ranging 119.1–395.4 nm and entrapment efficiency (%) ranging 47.6–79.6% were achieved.

**Figure 1:**
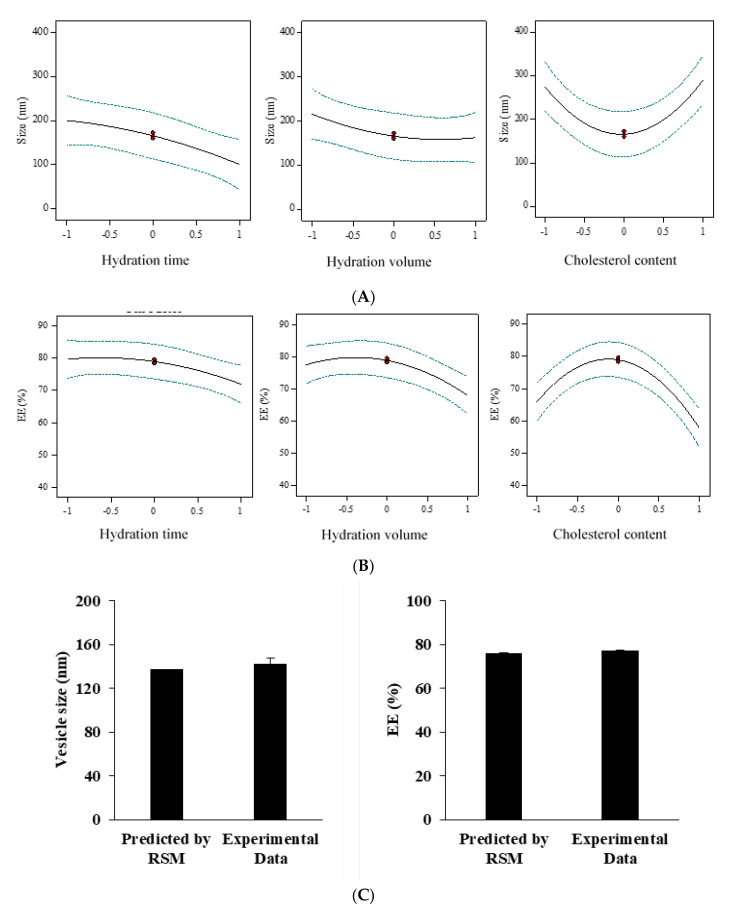
Box-Behnken method for average diameter (**A**) and encapsulation efficiency (EE) (**B**) as a function of the cholesterol content, hydration time, and hydration volume. The optimized responses thereof were in line with the experimental data (**C**), N = 3.

It was observed that the nanoparticle size decreased with increasing hydration time as well as the hydration volume (Figure 1A). In the case of cholesterol, as the amount of cholesterol increased, the size of the nanoparticles first decreased and then increased. The lowest size in 150 µmol of cholesterol (equal with level = 0) was obtained (Figure 1A). It was also found that with increasing the hydration time and volume, the entrapment efficiency decreased. With increasing the amount of cholesterol, the entrapment efficiency first increased and then decreased (Figure 1B). The optimal amount of entrapment efficiency with 150 μM cholesterol (equal with level = 0) was obtained (Figure 1B).

Other statistical patterns such as linear, 2FI, Quadratic, and cubic were also studied to find an association between various parameters and the nanoparticle size. Data fitting was implemented by the Design of Experiments software (Stat-East Inc., Minneapolis, MN, USA). The succeeding *p*-value of the quadratic pattern was found to be 0.0169 (significant) in the adjusted prototype for nanoparticle size (Supplementary Table S1). The lack-of-fit *p*-value was 0.0528 (not significant). The anticipated R-squared showed a slight (<0.2) difference from the perceived value, suggesting a proper fit (Supplementary Table S2). These conditions indicate that the quadratic model is suitable for describing niosomes size. Interpreting this response demanded no alteration with the approximate values of R-squared (R^2^), Standard deviation (SD), and coefficient of variation (%CV) listed in Supplementary Table S2, along with the regression equation formed for this retort.

Numerous statistical patterns such as linear, 2FI, Quadratic, and cubic were also probed for prototype inspection by applying the Design-Expert software to finding an association between the variables and entrapped efficiency. The sequential *p*-value of the quadratic form was found to be 0.0050 (significant) (Supplementary Table S3), with the lack-of-fit *p*-value of 0.1267 (not significant). The contrast in adjusted and predicted R-squared was <0.2, suggesting a good fit (Supplementary Table S4). These values suggest a quadratic model for the entrapped efficiency of niosomes. The quadratic and the comparative values of R^2^, SD, and %CV are provided in Supplementary Table S4, along with the generated regression equation. In the virtue of regression equations, a statement was expressed from the significance. The inverse impact of hydration time (A), hydration volume (B), and cholesterol content (C) on entrapment efficiency were explicitly revealed by the regression equation of the entrapped efficiency.

The size of niosomes can change with experimental conditions, such as cholesterol concentration, hydration time, and volume. Cholesterol, which is amphipathic, can immerse itself in a bilayer membrane with its hydrophilic head facing the water surface and the aliphatic chain line parallel to the hydrocarbon chains in the center of the bilayer [27]. It is known that cholesterol increases the chain order of the liquid-state bilayer and strengthens the nonpolar tail of the nonionic surfactant. At low cholesterol concentrations, it is expected that cholesterol led to close packing of surfactant monomers by increasing curvature and decreasing in size. However, increasing the cholesterol content leads to increased hydrophobicity of the bilayer membrane and may disrupt the vesicular membrane. Therefore, increasing the radius of the vesicles is a way to create a thermodynamically stable form. In addition, cholesterol can stabilize the structure of the bilayer by eliminating the peak phase transition temperature of the vesicles. As a result, it strengthens the two-layer structures and reduces the micro-fluidity of the bi-layer, a situation that would interfere with the size reduction during the sonication step [27].

The results also showed that the amount of EE% increased linearly with decreasing cholesterol concentration, which is consistent with previous studies, and likely due to the intercalation of cholesterol in the bilayer structure [28]. As cholesterol levels increase, hydrophobicity and stability of niosomes increase and permeability decreases, leading to the successful encapsulation of hydrophobic drugs into the bilayer structure of the vesicle. However, increased cholesterol can compete with the drug for encapsulation in the bilayer, hence excluding the drug as the amphiphiles assemble into vesicles [29]. Another study suggests that a decrease in EE% with an increase of cholesterol to a certain extent may be caused by the fact that an increase in cholesterol beyond a certain concentration can disrupt the linear structure of the vesicular membrane [30].

Furthermore, as the hydration time increases, the niosomal size decreases, which may be due to disruption of the vesicular structure and leakage of drug from the vesicles as the hydration time increases [31]. Studies showed that with an increase hydration volume, there is a decreasing trend in entrapment efficiency and particle size. This behavior can be explained by the possibility that increasing the hydration volume may increase drug leakage from the niosomes and lead to a reduction in entrapment and vesicle size [32]. Ruckmani et al. studied the effect of various variables, such as hydration time, sonication time, charge-inducing agent, centrifugation, and rotational speed of flask evaporation, on the amount of zidovudine loading in the niosome and its release. The results of their study showed that the encapsulation efficiency ranged from 72 to 80 [33]. Abdelbary et al. showed that the niosome formulated with a molar ratio of 1:1:0.1 of Tween 60, cholesterol, and dicetyl phosphate (DCP) had the highest EE level of 92% and release rate of 66% in 8 h [34].

The encapsulation efficiency and the niosomes’ size were contingent on the sort of surfactants and the quantity of cholesterol (i.e., lipid), as each trade in the chemical character and constitution instantly hit the hydrophilic–lipophilic balance (HLB) in the niosomal structure [35]. Among Span surfactants, Span 80 resulted in smaller niosomes, which can be assigned to an extension in the hydrophobic chain length of Span surfactant arrangement through Span 20 to Span 80, and also more hydrophobic–hydrophobic cooperation within the encapsulated *E. angustifolia*, cholesterol, DCP, and hydrophobic chain of surfactant [36]. It has previously been showed that the stearyl chain (C18) non-ionic surfactant offered more elevated entrapment efficiency than a lauryl chain (C12) non-ionic surfactant [37]. It was also confirmed that HLB was raised from 1.8 to 8.6 upon a drift through Span 85 to Span 20 [38]. Particle diameter and size are primary factors in novel drug delivery systems, which could affect encapsulation efficiency and drug release. It can be concluded that the amount of cholesterol could significantly alter the modest size of the vesicles. This outcome verifies other studies expressing a direct relationship between the lipid quantity and nano-vesicle size [39]. This result could be described by the cholesterol inclination to extend the bilayer unit as it has an insignificant impact on the charge at the bilayer surface and inter-bilayer separation [40].

As mentioned above, although the elevation of cholesterol enhanced the entrapment efficiency, an extra increment of the cholesterol content would dwindle entrapment efficiency due to the interruption of the bilayer structure [41]. It has been reported that the cholesterol/surfactant molar ratio of 1/1 reached the maximum entrapment efficiency [42]. The minuscule difference between anticipated and perceived responses verified the optimization procedure. Table 3 shows the assay for the optimized formula. The perceived response of the niosome size at 142.3 ± 5.1 and EE at 77.1% ± 0.3% was in line with the anticipated one, suggesting a proper optimization method (Figure 1C). Thus, the optimized formulation was applied for further empirical studies.

### 3.2. Characterization of Niosomes-Encapsulated E. angustifolia

#### 3.2.1. Morphological Studies

**Figure 2:**
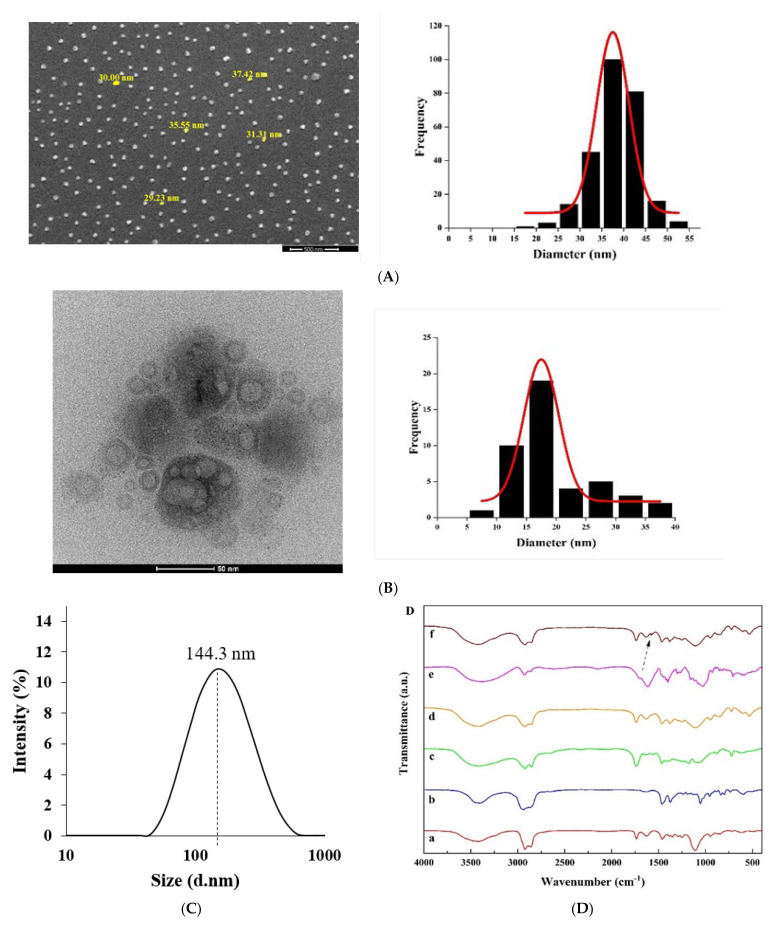
Morphological ascertainment of the optimized form. (**A**) Scanning electron microscopy (SEM), (**B**) transmission electron microscopy (TEM), (**C**) analysis of particle size distribution by dynamic light scattering (DLS), and (**D**) Fourier transform infrared (FTIR) spectra of (a) cholesterol, (b) Span 60, (c) Tween 60, (d) *E. angustifolia*, (e) Niosome, and (f) *E. angustifolia*-loaded niosome. Arrow: indicating the peak of *E. angustifolia*.

Morphological studies of the provided superlative niosomes were achieved by SEM and TEM. The eruption image area of the finest formulation can be observed, which proves identical globe-shaped morphology and stable surface with a mean dimension of 40 nm escorted by no niosomes’ convergence (Figure 2A). The inner configuration of niosomal *E. angustifolia*, assessed by TEM analysis, showed the rigid format of niosomes’ borders and the formation of globular niosomes (Figure 2B). Nanoparticles’ size captured by SEM and TEM reveals a more diminutive contrast to those measured by the Nano Zetasizer at 144.3 nm (Figure 2C). This inconsistency could be related to the drying procedure in SEM and TEM imaging. In other words, SEM and TEM measure the size of dried nanoparticles (i.e., the accurate diameter of nanoparticles), while DLS estimates the hydrodynamic diameter, compromising the core and any particle or molecule associated with the surface, including ions and water molecules. The different size measured by SEM and TEM was also reported previously. Mirzaie et al. synthesized various niosomes containing ciprofloxacin and found that the mean size measured by SEM was 57 nm, and 15 nm by TEM [43]. One of the reasons for this size difference could be the fusion of nanoparticles, which can only be detected by TEM [44].

#### 3.2.2. Fourier Transform Infrared (FTIR) Analysis

The FTIR spectra were studied to determine the chemical bonds formed in the niosomal systems. The optimum niosome formation in the absence of drug (i.e., empty niosome) exhibited the most featured peaks, which can be assigned to Span 60, Tween 60, and cholesterol (Figure 2D). Nonetheless, the C=C stretching (at 1674 cm^−1^) zenith of the cholesterol faded, validating the cholesterol enclosure in the lipid bilayer [45]. Further, the prominent peaks of the drug molecule (*E. angustifolia*) were also absent in the niosomes’ structures, which were observed by others as well [8,38].

#### 3.2.3. Kinetics and In Vitro Drug Release Studies

**Figure 3:**
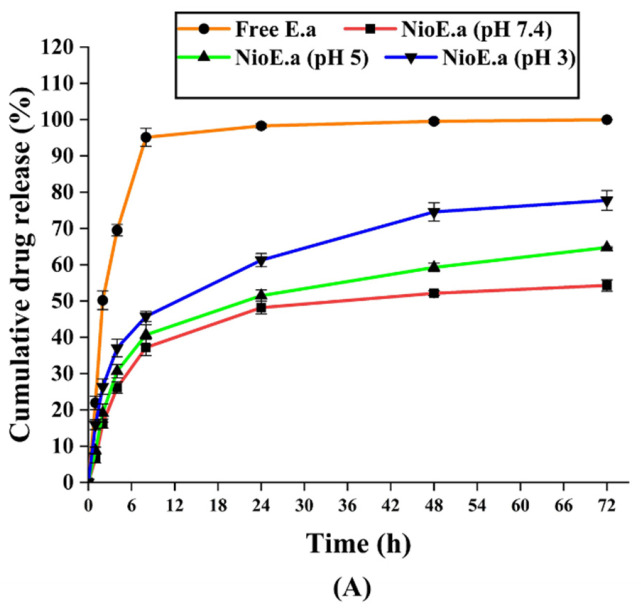

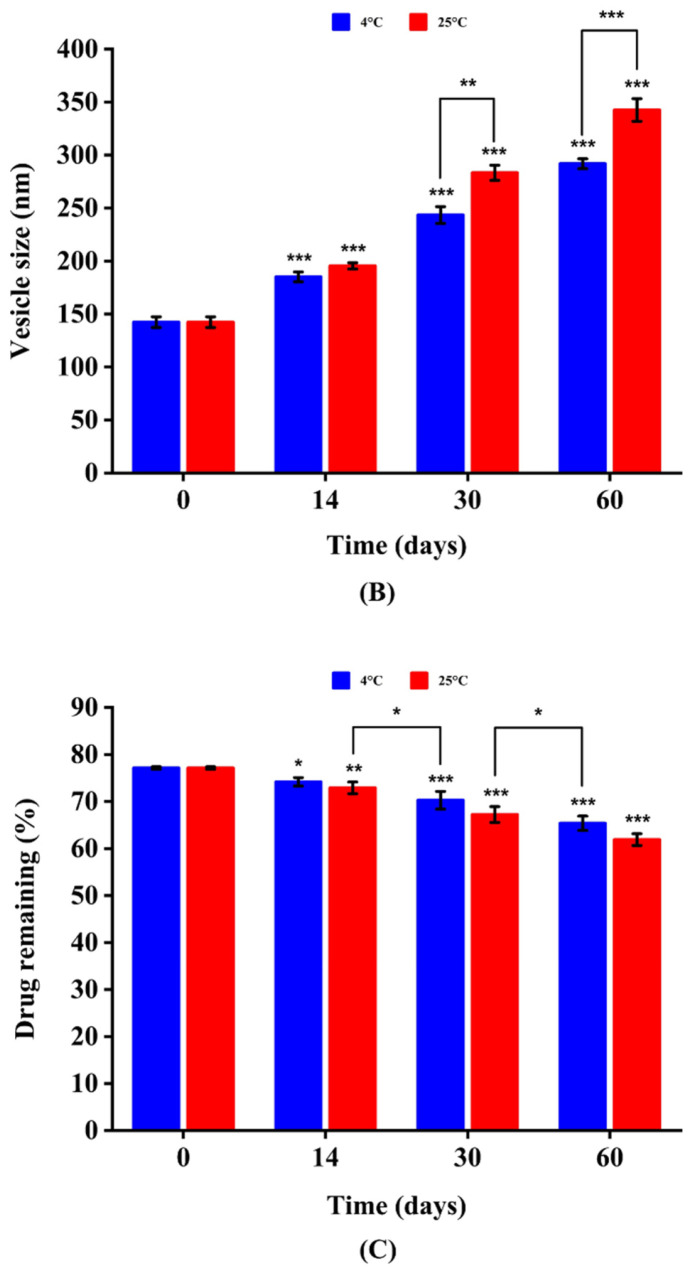
(**A**) In vitro drug release profile of *E. angustifolia* extracts at different pH from the optimized formulation of drug-loaded niosomes. Free Ea: free *E. angustifolia* extracts; NioEa (pH 3), NioEa (pH 5) and NioEa (pH 7.4): niosome-encapsulated *E. angustifolia* extracts kept at pH 3, pH 5, and pH 7.4, respectively. N = 3. Stability in respect to vesicle size (**B**) and Drug remaining (**C**) of optimum *E. angustifolia*-loaded niosomes during 60 days of storage at 4 ± 2 and 25 ± 2 °C. N = 3. ***: *p* < 0.001, **: *p* < 0.01, *: *p* < 0.05.

The release pattern of the *E. angustifolia* extract from the niosome was investigated at different pH values and for more than 72 h. The niosomal structures minimized the initial burst release dramatically compared to the free *E. angustifolia,* with the former releasing about 45–68% loaded drug after 24 h and the latter 95% after 8 h (Figure 3A). After 24 h, pH 3 and 5 allowed further release of 74% and 59% respectively, until 48 h, whereas pH 7.4 did not lead to further release and remained at 51%. The total 72 h drug release for the optimized formulation was 54%, 64%, and 78% at pH values of 7.4, 5, and 3, respectively. The *E. angustifolia* release profile determines an aggregated biphasic form, contrary to the free drug [39]. The release stage set about the hastened outline of the drug followed by a latent release stage. The rapid initial stage may be due to the free *E. angustifolia* excretion in the niosome and drug elution from the niosome-covering. The other stage is centrally linked to the dispersion of *E. angustifolia* throughout the bilayers [46]. Our results agreed with other studies, including an investigation by Namdeo and co-workers which proved an initial fast biphasic release of 5-fluorouracil from niosome followed by a passive one. Besides, a half 5-fluorouracil release rate from nanovesicles within 6 h was observed compared to the free drug release in 2 h [47].

Lower pH promoted a considerable increase of *E. angustifolia* drug release, likely due to swelling/breaking of niosomal structures at acidic pH [48]. Another reason for the pH-dependent response of the niosomal system could be the immense hydrolysis rate of surfactants (i.e., Span 60 and Tween 60) at acidic pH, which caused a break-out in drug molecules’ release at acidic conditions [49]. The lipid bilayer structure of niosomes assures that the entrapped *E. angustifolia* moves across the cell membrane, causing sustained release at physiological pH. Drug release and its crossing through a bilayer membrane depend on the fluidness and the structure of the bilayer membrane. In this regard, the electrostatic interaction between drugs and surfactants plays a decisive role, chiefly, under ionized conditions at a physiological pH [50]. This pH-dependent reaction plays a critical role in cancer cells, which habitually have acidic pH compared to healthy cells. The pH-dependent release can also be of advantage for treatment of dental carries, which is the direct consequence of the acidic pH microenvironments caused by bacteria and biofilm [51].

The *E. angustifolia* molecules’ release kinetics from niosome structure formed at non-identical pH were reviewed considering various kinetic models [52] (Supplementary Section SI-1). Studies show that the Korsmeyer–Peppas model is one of the most common models for drug release in niosomes, where the n parameter represents the drug release pathway [46,47]. Table 4 delineates the model parameters and the perception coefficient (R^2^) at all studied pH values. The data of release obeyed the Korsmeyer–Peppas kinetic model, with n = 0.4160 at pH = 7.4 (indicating the Fickian diffusion mechanism). In contrast, n grew at the acidic conditions and transfers to n > 0.45 (representing the Anomalous convey mechanism) [53]. Thus, the Korsmeyer–Peppas kinetic model fit the data best and was consequently used.

#### 3.2.4. Physical Stability of Niosomes-Encapsulated *E. angustifolia*

Previous studies have shown that niosomes can become swollen, swell/break downward, throughout the storage procedure due to the penetration of water molecules into the structure of the niosome [54]. In this study, the stability of the niosome at 25 °C (room temperature, RT) and 4 °C (fridge storage temperature) was investigated, in terms of size and drug release (Figure 3B,C).

The samples stored at 4 ± 2 °C were marginally more stable than those kept at 25 ± 2 °C, which could be attributed to the greater rigidity of the hydrophobic segment of niosomes at lower temperatures. The drug maintenance in niosomal formulation presents less than 20 percent of drug leakiness from the initially entrapped quantity of *E. angustifolia* at both circumstances. These outcomes agreed with other studies [32], where storage at low temperatures slightly prolonged the expiration duration of the niosomal structures.

The results presented in this study show that with increasing temperature, the amount of drug leakage from nanocarriers increases because the membranes of vesicles are more fluid at high temperatures [50]. Following the increase in fluidity, rupture and fusion of vesicles also increase, and due to the opening of the vesicular structures, the drug enclosed in them is released. In addition, at high temperatures, the structure of fatty acids in the nanocarrier membrane becomes irregular, which may reduce its thickness, thereby increasing drug release [55]. Studies have shown that nanoparticles increase in size during storage, which can be due to fusion and accumulation of vesicles. Further, the surface energy in nanocarriers depends on the size, so that smaller vesicles have higher surface energy and are more prone to melting [56].

### 3.3. Bioactivity of the Encapsulated Niosomes

To investigate whether the encapsulated niosomes possess more sustainable and higher antimicrobial activity compared to the free extracts, we tested the obtained niosomes against the clinical isolates of the multidrug-resistant *K. pneumonia*, listed by the WHO as one of the leading resistant pathogens.

#### 3.3.1. Isolation of *K. pneumoniae* Strains and Their Antibiotic Resistance Profile

Out of 100 clinical samples, 50 *K. pneumoniae* strains were recovered from clinical specimens based on microbiological tests. The disk diffusion antibiotic resistance test showed that 23 out of the 50 strains (46%) were multidrug-resistant (MDR), and all MDR strains were resistant to beta-lactam antibiotics (Supplementary Table S5). Moreover, all strains were sensitive to imipenem and colistin.

#### 3.3.2. Antibacterial Activity

The antimicrobial effects of niosome-encapsulated *E. angustifolia* extract, free extract, and free niosome were then determined against the *K. pneumoniae* isolates using the microdilution method. The MIC of the niosome-encapsulated extract was found to be 4 to 16 times lower than the free extract (Table 5), while drug-free niosomes exhibited no antibacterial activity. The MBC values were equal to MIC in some strains and higher than MIC in others. In the time-kill assay, microbial growth was inhibited after treatment with niosome-encapsulated *E. angustifolia* extract, to a similar degree to that treated with free extract for 8 h. Moreover, the results also showed that prolonged incubation for 24 h allowed slightly higher inhibition activity for the niosome-encapsulated extracts, compared to the free extracts (Supplementary Table S6). The reason for higher antibacterial activity of the encapsulated niosome than that of the free extract could be due to fusion of niosomes with the bacterium cell membrane, giving rise to its direct interaction with the cell membrane and facilitating the extract release to the local environment. Indeed, fusion of the niosome structure and the outer membrane of the bacterium has been reported to increase the antimicrobial effects [57]. This may increase the fluidity of the bacterial membrane and facilitate the entry of the released extract into the bacterial cell. Moreover, niosomes can increase the stability of the extract, thus providing prolonged activity [58]. The greater penetration of the extract-encapsulated niosome into the bacterial cells can lead to higher bactericidal activities [59]. Additionally, Patel et al. reported the significant antimicrobial activity of propolis-loaded niosomes against *Staphylococcus aureus* [60].

#### 3.3.3. Cell Toxicity Assay

**Figure 4:**
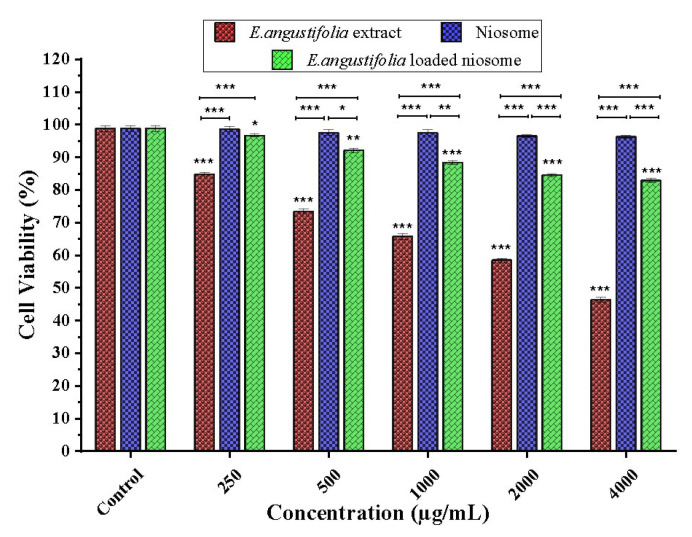
Cytotoxicity of various concentrations of *E. angustifolia* extract and *E. angustifolia*-loaded niosome against HFF normal cells within 72 h. The viable HFF cells subjected to distilled water were used as controls and set as 100%. The cytotoxic cut-off was set to 70% viable cells, i.e., a lethal dose of 30%. N = 3. ***: *p* < 0.001, **: *p* < 0.01, *: *p* < 0.05.

To evaluate the cytotoxicity of niosome-encapsulated *E. angustifolia*, free extract, and non-loaded niosome, human foreskin fibroblasts (HFF) were tested using the MTT method. The results showed that free niosome up to 4000 ug mL^−1^ had no significant cytotoxic effects at the tested concentrations. The encapsulated extract exhibited reduced cytotoxic effects compared to the extract, namely, the latter showed cytotoxicity at the concentrations above 250 ug mL^−1^ and the former did not exhibit cytotoxicity even at concentration of 4000 ug mL^−1^ (Figure 4). The non-toxicity of free niosomes is highly desirable for nanocarriers. Furthermore, the slow release of extract from niosomes can explain the lower toxicity of the encapsulated extract compared to the free extract [61].

## 4. Conclusions

Here, niosomes were successfully prepared via the Design of Experiment approach for delivery of bioactive plant extracts. The obtained niosomes led to enhanced stability and antimicrobial activity compared to free extracts, allowing high encapsulation efficiency and controlled release. The resulting niosomes also showed activity towards multidrug-resistant bacterial strains and exhibited lower cytotoxic effects relative to the extracts. This study contributes to the treatment strategies for infectious diseases caused by multidrug-resistant bacteria through applying nature-derived drugs. Further studies are needed to identify the active compounds of the plant extracts and to study the underlying mechanism. We see an opportunity to extend the knowledge gained here surrounding niosomes and plant extracts into other bioactive components for improved stability and antibacterial activity. While the therapeutic efficacy of pathogen elimination in reality remains to be demonstrated in clinics, the proposed niosome-based drug release is thought to help reduce the overuse of antibiotics by allowing nature-derived materials and controlled drug release.

## Figures and Tables

**Table 1:** Different levels for variables in the Box-Behnken optimization.

Level	−1	0	+1
A (Hydration time, min)	30	45	60
B (Hydration volume, mL)	6	8	10
B (Hydration volume, mL)	6	8	10

**Table 2:** Design of Experiments using the Box-Behnken method to optimize the niosomal formulation of *E. angustifolia* extract. Total lipid (Span 60, Tween 60, and Cholesterol) concentration: 300 µmol; ratio of Span 60 and Tween 60 set to 1 to 1 (molar ratio); ratio of surfactant and Cholesterol set to 1 to 1 (molar ratio).

Run	Levels of Independent Variables			Dependent Variables		
	Hydration Time (min)	Hydration Volume (mL)	Cholesterol Content (µmol)	Average Size (nm)	Polydispersity Index (PDI)	Entrapment Efficiency (EE) (%)
1	0	−1	−1	327.1	0.37	67.2
2	0	0	0	285.4	0.31	49.6
3	0	1	−1	259.6	0.32	52.2
4	−1	0	−1	350.1	0.38	54.2
5	0	1	1	291.3	0.34	69.7
6	1	1	0	119.1	0.23	64.0
7	1	0	1	181.9	0.18	73.3
8	0	−1	1	395.4	0.39	61.3
9	−1	1	0	172.5	0.26	79.6
10	−1	−1	0	235.8	0.30	56.4
11	0	0	0	188.7	0.23	66.1
12	1	0	−1	164.8	0.26	78.8
13	1	−1	0	145.7	0.24	47.6
14	−1	0	1	160.2	0.27	78.3
15	0	0	0	205.7	0.28	75.4

**Table 3:** Desirability criteria and predicted values for the variables.

Number	A (Hydration Time, min)	B (Hydration Volume, mL)	C (Cholesterol Content, µmol)	Desirability
1	53	8	150	0.933

**Table 4:** The kinetic release models and the parameters obtained for optimum niosomal formulation. * Diffusion or release exponent; ** Free *E. angustifolia;* *** *E. angustifolia*-loaded niosome.

Release Model	Zero-Order	Korsmeyer–Peppas		First-Order	Higuchi
	R^2^	R^2^	n *	R^2^	R^2^
FreeEa **—pH 7.4	0.8109	0.8425	0.5944	0.9254	0.7468
NioEa ***—pH 7.4	0.5981	0.9925	0.4160	0.7548	0.9322
NioEa—pH 5	0.6541	0.9739	0.4675	0.7628	0.9691
NioEa—pH 3	0.7739	0.9785	0.5146	0.8035	0.9475

**Table 5:** MIC and MBC values of selected bacteria and niosome-encapsulated *E. angustifolia* extract.

Strain No.	MIC of *Ea* Extract (µg mL^−1^)	MIC of Extract-Loaded Niosome (µg mL^−1^)	Increased Efficacy of Niosome (Fold)	MBC of *Ea* Extract (µg mL^−1^)	MBC of Extract-Loaded Niosome (µg mL^−1^)	Increased Efficacy of Niosome (Fold)
4	2000	125	16.0	4000	250	16.0
6	4000	500	8.0	4000	500	8.0
10	2000	125	16.0	2000	125	16.0
13	2000	500	4.0	2000	500	4.0
16	4000	250	16.0	4000	250	16.0
24	2000	250	8.0	2000	250	8.0
29	1000	125	8.0	2000	125	16.0
33	2000	125	16.0	2000	250	8.0
37	4000	1000	4.0	4000	2000	2.0
46	1000	62.5	16.0	1000	125	8.0
51	2000	500	4.0	4000	1000	4.0
56	1000	62.5	16.0	2000	125	16.0
61	1000	125	8.0	1000	125	8.0
66	1000	250	4.0	1000	250	4.0
71	500	62.5	8.0	500	125	4.0
73	1000	62.5	16.0	1000	125	8.0
77	2000	125	16.0	2000	125	16.0
82	2000	250	8.0	2000	250	8.0
84	1000	125	8.0	1000	250	4.0
87	2000	250	8.0	2000	250	8.0
91	1000	62.5	16.0	1000	125	8.0
94	2000	250	8.0	2000	250	8.0
96	1000	250	4.0	1000	250	4.0

## Data Availability

The data presented in this study are available upon request from the corresponding author. The data are not publicly available due to privacy restrictions.

## Supplementary Materials

The following are available online at https://www.mdpi.com/article/10.3390/nano11061573/s1, Table S1: Analysis of variance for the quadratic polynomial model for size. S: significant; NS: not significant., Table S2: Analysis of variance for the quadratic polynomial model for EE. S: significant; NS: not significant. Table S3: Regression analysis for response size for fitting to the quadratic model. Table S4: Summary of results of regression analysis for responses EE, for fitting to the quadratic model. Table S5: Antibiotic resistance profile among MDR strains of *K. pneumoniae*. Table S6: The CFU/ml of *K. pneumoniae* strains after treatment with sub-MIC concentration of free *E. angustifolia* extract and niosome encapsulated extract.
