# Supplementary material for: Enhanced Antibacterial Activity of Echinacea angustifolia Extract against Multidrug-Resistant Klebsiella pneumoniae through Niosome Encapsulation
Source: Nanomaterials (Basel). 2021 Jun 15;11(6):1573. doi: 10.3390/nano11061573 (PMC8232788; doi:10.3390/nano11061573)
Supplement: Supplementary file 1 [file nanomaterials-11-01573-s001.zip › nanomaterials-1214515-supplementary.pdf]

Supporting Information

Enhanced Antibacterial Activity of *Echinacea angustifolia* Extract against Multidrug Resistant *Klebsiella pneumoniae* through Niosome Encapsulation

Maryam Moghtaderi [1], Amir Mirzaie [2,*], Negar Zabet [3], Ali Moammeri[1], Amirreza Mansoori-Kermani[4], Iman Akbarzadeh [4,*], Faten Eshrati Yeganeh [5], Arman Chitgarzadeh [6], Aliasghar Bagheri Kashtali [6] and Qun Ren [7,*]

[1] School of Chemical Engineering, College of Engineering, University of Tehran, Tehran, Iran

[2] Department of Biology, Parand Branch, Islamic Azad University, Parand, Iran

[3] Department of Medicinal Chemistry, School of Pharmacy, Shahid Beheshti University of Medical Sciences, Tehran, Iran

[4] Department of Chemical and Petrochemical Engineering, Sharif University of Technology, Tehran, Iran

[5] Department of Chemistry, Science and Research Branch, Islamic Azad University, Tehran, Iran

[6] Department of Biology, Roudehen Branch, Islamic Azad University, Roudehen, Iran

[7] Laboratory for Biointerfaces, Empa, Swiss Federal Laboratories for Materials Science and Technology, 9014 St. Gallen, Switzerland

SI-1. Kinetic models

The explanation of each kinetic model used in this study is as follow[1]:

- ***Zero-order model:*** $C_t = C_0 + K_0 t$

where C_t represents the amount of drug released at time t, C_0 is the initial concentration of drug released which is generally zero. In this model, the release process takes place at a constant rate and it is independent of initial drug concentration.

- ***First-order model:*** $Log\ C = LogC_0 - Kt/2 \cdot 303$

Where C_0 is the initial concentration of the drug, k is the first-order rate constant, and t is the time. C is the drug remaining in the carrier at time t. Log C and t have s linear relationship and K/2.303 is the slope of the straight line. This model can be used to describe water-soluble drugs in porous matrices.

- ***Higuchi model:*** $Q = K_H \sqrt{t}$

Where, K_H is the Higuchi constant and it is obtained from the slope of the line. The data obtained were plotted as cumulative percentage drug release versus square root of time. This model can be useful in the case of matrix tablets containing water-soluble drugs.

- ***Korsmeyer-Peppas model:*** $M_t/M_\infty = Kt^n$

Linear Form: logM=Log K+n Logt

Where M_t/M_0 is a fraction of drug released at time t, k is the release rate constant and n is the release exponent. The n value is used to characterize different releases for cylindrical shaped matrices. For the case of spherical tablets:

- n ≤ 0.43: Fickian diffusion mechanism
- 0.43 < n <0.85: non-Fickian transport.
- n = 0.85: Case II (relaxational) transport.
- n > 0.85: super case II transport.

The first 60% drug release data were fitted in this model. Data obtained from drug release studies were plotted as log cumulative percentage drug release versus log time. This model is suitable for polymeric systems.

Reference:

[1] S. Dash, P.N. Murthy, L. Nath, P. Chowdhury, Acta Pol Pharm, 67 (2010) 217-223.

Table S1. Analysis of variance for the quadratic polynomial model for size. S: significant; NS: not significant.

Source	Sum of squares	Degree of Freedom	Mean square	F-Value	P-Value	Evaluation
Model	89137.47	9	9904.16	8.01	0.0169	S
A	19860.25	1	19860.25	16.07	0.0102	S
B	5618.00	1	5618.00	4.55	0.0862	NS
C	492.98	1	492.98	0.40	0. 5554	NS
AB	524.41	1	524.41	0.42	0.5435	NS
AC	9428.41	1	9428.41	7.63	0.0397	S
BC	1.96	1	1.96	1.586E-003	0.9698	NS
A^2	857.75	1	857.75	0.69	0.4428	NS
B^2	1997.35	1	1997.35	1.62	0.2596	NS
C^2	50077.08	1	50077.08	40.52	0.0014	S
Residual	6179.25	5	1235.85			

Table S2. Analysis of variance for the quadratic polynomial model for EE. S: significant; NS: not significant.

Source	Sum of squares	Degree of Freedom	Mean square	F-Value	P-Value	Evaluation
Model	1628.91	9	180.99	13.77	0.0050	S
A	121.76	1	121.76	9.26	0.0286	S
B	182.98	1	182.98	13.92	0.0136	S
C	134.89	1	134.89	10.26	0.0239	S
AB	2.274E-013	1	2.274E-013	1.730E-014	1.0000	NS
AC	0.027	1	0.027	2.071E-003	0.9655	NS
BC	27.04	1	27.04	2.06	0.2110	NS
A^2	36.22	1	36.22	2.76	0.1578	NS
B^2	137.60	1	137.60	10.47	0.0231	S
C^2	1068.59	1	1068.59	81.29	0.0003	S
Residual	65.73	5	13.15			

Table S3. Regression analysis for response size for fitting to the quadratic model.

Quadratic model	R^2	Adjusted R^2	Predicted R^2	SD	%CV
	0.9352	0.8185	0.2610	35.15	15.14

Regression equations of the fitted model:

$$\text{Particle Size} = +165.83 - 49.83 * A - 26.50 * B + 7.85 * C - 11.45 * A * B - 48.55 * A * C + 0.70 * B * C - 15.24 * A^2 + 23.26 * B^2 + 116.46 * C^2$$

where A is Hydration time, B is Hydration volume and C is Cholesterol content according to the normalized data between -1 and 1.

Table S4. Summary of results of regression analysis for responses EE, for fitting to the quadratic model.

Quadratic model	R^2	Adjusted R^2	Predicted R^2	SD	%CV
	0.9612	0.8914	0.3868	3.63	5.58

Regression equations of the fitted model:

EE = +78.93- 3.90 * A- 4.78* B- 4.11 * C- 0.082* A * C +2.60* B * C- 3.13 * A2- 6.10 * B2- 17.01 * C2

where A is Hydration time, B is Hydration volume and C is Cholesterol content according to the normalized data between -1 and 1

Table S5. Antibiotic resistance profile among MDR strains of *K. pneumoniae*. CAZ: Ceftazidime, C: Chloramphenicol, GN: Gentamicin, TOB: Tobramycin, TE: Tetracycline, AMP: Ampicillin, CIP: Ciprofloxacin, AMC: Amoxicillin/clavulanic acid, NA: Nalidixic acid.

Strain NO.	Resistance pattern	MDR
6, 24, 37, 51	AMP, AMC, CAZ, CIP, TOB	+
4, 13, 71, 82, 96	AMP, AMC, TE, AK, NA	+
33, 46, 61, 77, 84	AMP, AMC, GN, C, TE	+
10, 29, 66, 91, 94	AMP, AMC, CAZ, CIP, TE	+
16, 73, 87, 56	AMP, AMC, TOB, CIP, GN	+

Table S6. The CFU/ml of *K. pneumoniae* strains after treatment with sub-MIC concentration of free *E. angustifolia* extract and niosome encapsulated extract.

Isolate NO.	0 h	2 h<br>free extract/<br>niosome encapsulated extract	4 h<br>free extract/<br>niosome encapsulated extract	8h<br>free extract/<br>niosome encapsulated extract	24 h<br>free extract/<br>niosome encapsulated extract
**4**	5×10^5	5.3×10^5<br>5.1×10^5	5.3×10^5<br>5.1×10^5	5.5×10^5<br>5.2×10^5	6.1×10^5<br>$5.5\ \times10^5$
**6**	5×10^5	5.2×10^5<br>5.1×10^5	5.2×10^5<br>5.1×10^5	5.4×10^5<br>5.2×10^5	5.8×10^5<br>5.3×10^5
**10**	5×10^5	5.3×10^5<br>5.1×10^5	5.3×10^5<br>5.1×10^5	5.8×105<br>5.2×105	$6.2\ \times10^5$<br>5.3×10^5
**13**	5×10^5	5.3×10^5<br>5.1×10^5	5.3×10^5<br>5.1×10^5	5.6×10^5<br>5.3×10^5	6.1×10^5<br>5.4×10^5
**16**	5×10^5	5.2×10^5<br>5.1×10^5	5.2×10^5<br>5.1×10^5	5.9×10^5<br>5.3×10^5	6.3×10^5<br>5.4×10^5
**24**	5×10^5	5.3×10^5<br>5.1×10^5	5.3×10^5<br>5.1×10^5	6.1×10^5<br>5.3×10^5	6.3×10^5<br>5.5×10^5
**29**	5×10^5	5.2×10^5<br>5.1×10^5	5.2×10^5<br>5.1×10^5	5.9×10^5<br>5.3×10^5	6.3×10^5<br>5.4×10^5
**33**	5×10^5	5.3×10^5<br>5.1×10^5	5.3×10^5<br>5.1×10^5	6.1×10^5<br>5.3×10^5	6.3×10^5<br>5.5×10^5
**37**	5×10^5	5.3×10^5<br>5.1×10^5	5.3×10^5<br>5.1×10^5	5.6×10^5<br>5.3×10^5	6.1×10^5<br>5.4×10^5
**46**	5×10^5	5.2×10^5<br>5.1×10^5	5.3×10^5<br>5.1×10^5	5.6×10^5<br>5.3×10^5	6.1×10^5<br>5.4×10^5
**51**	5×10^5	5.2×10^5<br>5.1×10^5	5.2×10^5<br>5.1×10^5	5.4×10^5<br>5.2×10^5	5.8×10^5<br>5.3×10^5
**56**	5×10^5	5.3×10^5<br>5.1×10^5	5.3×10^5<br>5.1×10^5	5.6×10^5<br>5.3×10^5	6.1×10^5<br>5.4×10^5
**61**	5×10^5	5.2×10^5<br>5.1×10^5	5.2×10^5<br>5.1×10^5	5.9×10^5<br>5.3×10^5	6.3×10^5<br>5.4×10^5
**66**	5×10^5	5.2×10^5<br>5.1×10^5	5.2×10^5<br>5.1×10^5	5.4×10^5<br>5.2×10^5	5.8×10^5<br>5.3×10^5
**71**	5×10^5	5.2×10^5<br>5.1×10^5	5.2×10^5<br>5.1×10^5	5.9×10^5<br>5.3×10^5	6.3×10^5<br>5.4×10^5
**73**	5×10^5	5.2×10^5<br>5.1×10^5	5.2×10^5<br>5.1×10^5	5.9×10^5<br>5.3×10^5	6.3×10^5<br>5.4×10^5
**77**	5×10^5	5.3×10^5<br>5.1×10^5	5.3×10^5<br>5.1×10^5	6.1×10^5<br>5.3×10^5	6.3×10^5<br>5.5×10^5
**82**	5×10^5	5.2×10^5<br>5.1×10^5	5.2×10^5<br>5.1×10^5	5.9×10^5<br>5.3×10^5	6.3×10^5<br>5.4×10^5
**84**	5×10^5	5.2×10^5<br>5.1×10^5	5.2×10^5<br>5.1×10^5	5.9×10^5<br>5.3×10^5	6.3×10^5<br>5.4×10^5
**87**	5×10^5	5.3×10^5<br>5.1×10^5	5.3×10^5<br>5.1×10^5	5.5×10^5<br>5.2×10^5	6.1×10^5<br>$5.5\ \times10^5$
**91**	5×10^5	5.2×10^5<br>5.1×10^5	5.2×10^5<br>5.1×10^5	5.9×10^5<br>5.3×10^5	6.3×10^5<br>5.4×10^5
**94**	5×10^5	5.2×10^5	5.2×10^5	5.4×10^5	5.8×10^5

		5.1×10^5	5.1×10^5	5.2×10^5	5.3×10^5
**96**	5×10^5	5.3×10^5<br>5.1×10^5	5.3×10^5<br>5.1×10^5	5.5×10^5<br>5.2×10^5	6.1×10^5<br>5.5×10^5
